# Supplementary material for: Saccharomyces boulardii (CNCM I-745) improves intestinal damage in sepsis by remodeling intestinal flora structure
Source: Front Cell Infect Microbiol. 2025 Jul 31;15:1602792. doi: 10.3389/fcimb.2025.1602792 (PMC12350329; doi:10.3389/fcimb.2025.1602792)
Supplement: Supplementary file 1 [file DataSheet1.zip › summary/summary/6_Taxonomy_community/1_Taxonomy_profiling/5_Family/All/Family_abund_top30.sample.cluster.pdf]

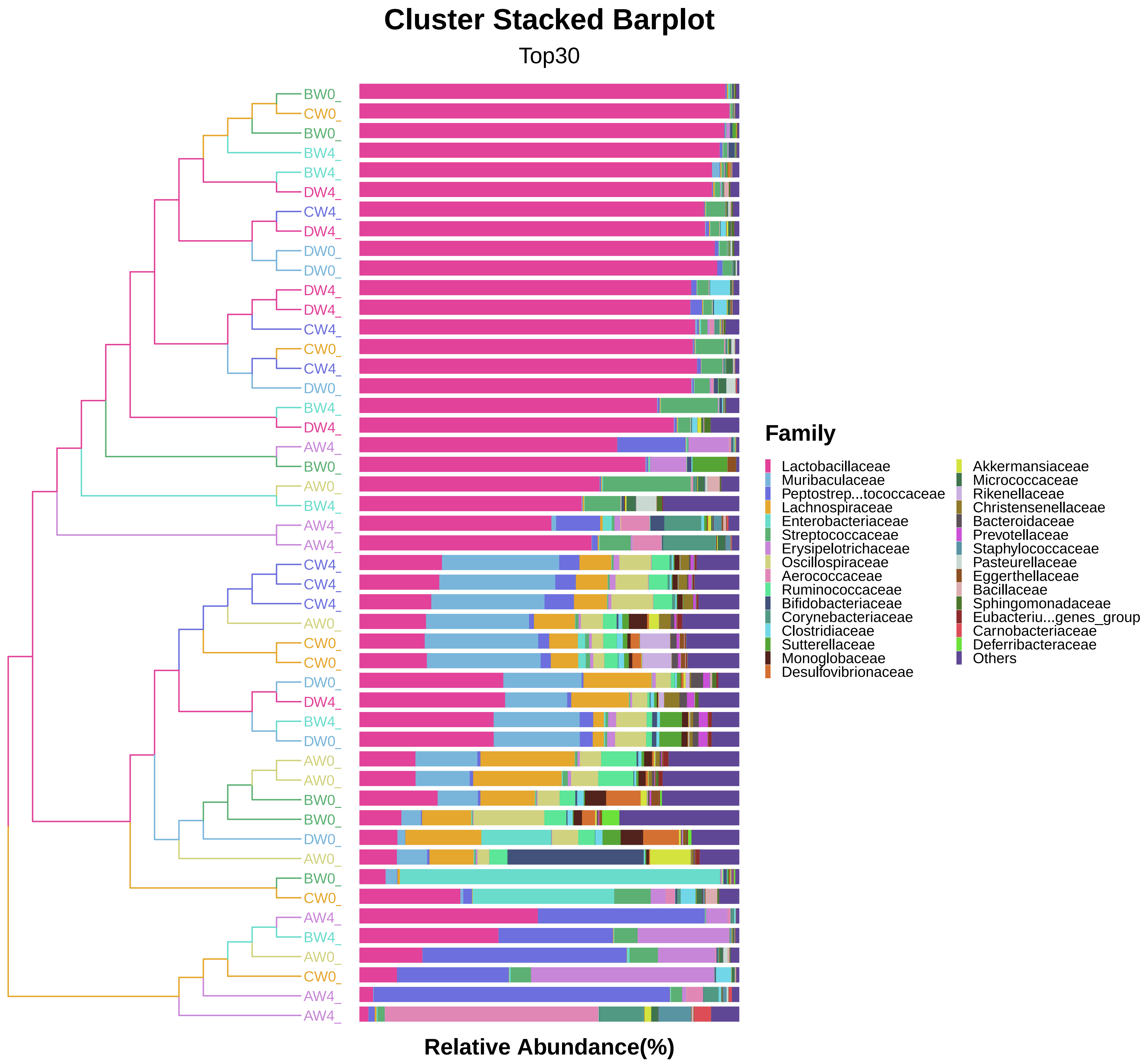
Cluster Stacked Barplot
Top30
BW0_
CW0_
BW0_
BW4_
BW4_
DW4_
CW4_
DW4_
DW0_
DW0_
DW4_
DW4_
CW4_
CW0_
CW4_
DW0_
BW4_
DW4_
AW4_
BW0_
AW0_
BW4_
AW4_
AW4_
CW4_
CW4_
CW4_
AW0_
CW0_
CW0_
DW0_
DW4_
BW4_
DW0_
AW0_
AW0_
BW0_
BW0_
DW0_
AW0_
BW0_
CW0_
AW4_
BW4_
AW0_
CW0_
AW4_
AW4_
Relative Abundance(%)
Family
Lactobacillaceae
Muribaculaceae
Peptostrep...tococcaceae
Lachnospiraceae
Enterobacteriaceae
Streptococcaceae
Erysipelotrichaceae
Oscillospiraceae
Aerococcaceae
Ruminococcaceae
Bifidobacteriaceae
Corynebacteriaceae
Clostridiaceae
Sutterellaceae
Monoglobaceae
Desulfovibrionaceae
Akkermansiaceae
Micrococcaceae
Rikenellaceae
Christensenellaceae
Bacteroidaceae
Prevotellaceae
Staphylococcaceae
Pasteurellaceae
Eggerthellaceae
Bacillaceae
Sphingomonadaceae
Eubacteriu...genes_group
Carnobacteriaceae
Deferribacteraceae
Others
